# Predictors of Early Mortality for Mechanically Ventilated Spontaneous Intracerebral Hemorrhage Patients

**DOI:** 10.7759/cureus.27935

**Published:** 2022-08-12

**Authors:** Kati K Reddy, Carmela San Luis, Parul Goyal, Kareem Elzamly, Tanvir Rizvi, Anand Mamdani, Christa Nobleza

**Affiliations:** 1 Neurology, University of Mississippi Medical Center, Jackson, USA; 2 Neurology, Guthrie Medical Group, Corning, USA; 3 Epilepsy and Clinical Neurophysiology, Baylor College of Medicine, Houston, USA; 4 Neurology, Tulane University School of Medicine Center for Clinical Neurosciences, New Orleans, USA; 5 Radiology and Medical Imaging, University of Virginia Health, Charlottesville, USA; 6 Neurology, Baptist Health Memphis, Memphis, USA

**Keywords:** mortality indicators, hemorragic stroke, prolonged mechanical ventilation, spontaneous hemorrhage, neurology and neurosurgery

## Abstract

Introduction

Spontaneous intracerebral hemorrhage (sICH) carries a high mortality burden. Limited data are available on early mortality (EM) and sICH. This study attempted to identify the independent predictors of EM and analyze the mortality characteristics for mechanically ventilated patients with sICHs at a tertiary care hospital over a period of five years.

Methods

An Institutional Review Board (IRB)-approved and Health Insurance Portability and Accountability Act (HIPPA)-compliant retrospective analysis was performed on sICH patients admitted at the University of Mississippi Medical Center Neuroscience Intensive Care Unit between January 1, 2013, to December 31, 2017. Patients were divided into two cohorts: EM cohort (death within seven days of admission) versus survivor cohort (alive more than seven days after admission). Demographic, comorbidity, clinical, and radiographic data were collected for each patient. Outcomes were compared utilizing student t-test or Mann-Whitney U tests for continuous variables. Logistic regression analysis was performed to determine independent predictors of EM.

Results

A total of 204 mechanically ventilated patients with sICHs, with a mean age of 59.73 (SD ±14.30), mostly African American (137, 67%), were included in the study. The characteristics of the two cohorts were comparable except the EM cohort had a lower proportion of patients with hypertension and end-stage renal disease; lower median Glasgow Coma Score (GCS) on admission; lower proportion of surgical evacuation and external ventricular drain (EVD) placement; higher proportion of lobar hemorrhage, brainstem involvement, midline shift, hydrocephalus, intraventricular hemorrhage component, and right-sided intracerebral hemorrhage (ICH); higher median ICH score; and higher ICH volume compared to the survivor cohort.

Overall, the mortality of mechanically ventilated sICH patients in this institution was 53% (N=109), with 47% (N=96) not surviving beyond seven days.

Logistic regression analysis revealed that ICH volume and brainstem involvement increased the odds of EM, while a history of hypertension, surgical evacuation, and EVD placement decreased the odds of EM.

Conclusions

This study on mechanically ventilated sICH patients identified ICH volume and brainstem involvement as independent predictors of increased EM. History of hypertension, EVD placement, and surgical evacuation decreased its odds. Further studies should be conducted to explore potentially modifiable processes that can improve patient outcomes, most importantly EM, especially in this cohort of patients.

## Introduction

Intracerebral hemorrhage (ICH) is the second most common subtype of stroke that can lead to severe disability or death. In the United States, it accounts for 8-15% of all strokes [1]. Primary intraparenchymal hemorrhage, another term for spontaneous intracerebral hemorrhage (sICH), is due to the rupture of damaged small arteries or arterioles, most commonly due to hypertension or cerebral amyloid angiopathy [2]. Despite advances in medical science, sICH remains to be the deadliest, most debilitating, and least treatable type of stroke [3]. No treatment has shown a proven advantage in improving mechanically ventilated (MV) patient outcomes after sICH, minimizing either mortality or long-term disability burden [3]. Trials on reducing blood pressure, osmotherapy, and ultra-early hemostatic therapy, and surgical evacuation of sICH hematoma have inconsistent effects on mortality and functional outcome [3]. Furthermore, no generally accepted early prognostic model or grading scale for MV sICH patients is currently used for routine triage and acute intervention, whether as part of clinical care or study [3]. In clinical practice, prognostic scoring systems have limitations for end-of-life decision-making. Few studies have been conducted to identify predictors of early mortality (EM) in MV patients with sICH [2].

A retrospective analysis of 72 sICH patients reported that 49% patients progressed to brain death, 39% died after palliative extubation, and 12% were discharged alive [4]. They showed the absence of corneal reflexes and the presence of the “swirl” sign on the initial computed tomography (CT) scan of the head [4]. Studies have suggested that identifying the clinical and radiographic predictors of EM and poor patient outcomes is invaluable in identifying and triaging high-risk patients appropriately and directing decision-making [5,6]. There are only a few studies analyzing predictors of EM among MV sICH patients.

The aim of this study was to determine the proportion of MV patients with sICH who expired before seven days (EM) and to subsequently identify the independent predictors of EM.

The data were presented in the University of Mississippi Medical Center (UMMC) Medical Student Research Program poster session in July 2020.

## Materials and methods

Study design

The University of Mississippi Medical Center (UMMC) Institutional Review Board (IRB) approved these investigations (IRB 2018-0009). As this is a retrospective study, the written consent requirement was waived.

Patient selection

All adult MV patients with sICH admitted into the UMMC Neuroscience Intensive Care Unit (NSICU) between January 1, 2013, and December 31, 2017, were screened for inclusion. A total of 238 MV patients with sICH were identified. Patients with anti-coagulation-associated hemorrhages and brain mass/tumor-associated hemorrhages were excluded, as well as patients with incomplete data. A total of 204 MV sICH patients were included in this study.

Data collection

Demographic, clinical, and radiographic data were collected for each patient utilizing electronic medical records. Radiographic characteristics were reviewed by a neuroradiologist (T.R.). ICH volume was based on the ABC/2 method [7].

Surgical intervention decision-making

In our institution, surgical intervention for sICH includes external ventricular drain (EVD) placement or surgical evacuation of the hematoma or decompressive craniectomy. For the purpose of this study, “surgical intervention” refers to evacuation of hematoma and decompressive craniectomy, while EVD placement is specified as such. The decision for EVD placement or surgical intervention is discussed among the family, neurocritical care team, and the neurosurgery team. There is no institutional protocol followed to dictate surgical intervention or EVD placement.

Data analysis

For this study, EM was defined as patient death within seven days of admission [8]. Patients were grouped according to the EM cohort (death within seven days of admission) versus “survivor” cohort (alive more than seven days after admission).” Characteristics were compared utilizing Student’s t-test or Mann-Whitney U tests for continuous variables. Chi-square tests were utilized for categorical variables as applicable. Descriptive statistics were used for the characterization of the overall study population. Factors showing a significant univariate relationship with EM were entered into a multivariate logistic regression to identify independent predictors of EM. Significance level was set at p=0.05 for all analyses. SPSS Version 24 (IBM Corp., Armonk, NY) was utilized for statistical analysis.

## Results

A total of 204 MV patients with sICH, with a mean age of 59.73 (SD ±14.30), mostly African American (137, 67%), were included in the study. The characteristics of the two cohorts were comparable (Table 1). It was found that among MV sICH patients, the overall mortality was 53% (N=109), with EM occurring in 47% (N=96) (Table 1).

**Table 1:** Overall characteristics of mechanically entilated patients with spontaneous intracerebral hemorrhage *Missing 20 patients’ BMI data. BMI, body mass index; CC, cubic centimeters; COPD, chronic obstructive pulmonary disease; ESRD, end-stage renal disease; GCS, Glasgow Coma Score; ICH, intracerebral hemorrhage; ICU, intensive care unit; LOS, length of stay; SOFA, sequential organ function assessment

Patient characteristics	N=204
Demographic characteristics	
Age (in years), mean (±SD)	59.73 (±14.30)
Age > 80, n (%)	22 (10)
Female sex, n (%)	102 (50)
Race, n (%)	
African American	137 (67.2)
Caucasian	64 (31.4)
Asian	1 (0.5)
Hispanic	1 (0.5)
Native American	1 (0.5)
Unknown	1 (0.5)
Charlson Comorbidity index, mean (±SD)	2.18 (±1.68)
Charlson Comorbidity index, median (IQR)	2 (1-3)
Comorbidity, n (%)	
Stroke	4 (2)
Intracerebral hemorrhage	4 (2)
Hypertension	153 (75)
Coagulopathy	21 (10)
Diabetes	53 (26)
COPD	3 (1.5)
ESRD	28 (13.7)
Admission clinical characteristics	
GCS on admission, mean (±SD)	7 (±4.37)
GCS on admission, median (IQR)	6 (3-10)
SOFA, mean (±SD)	4.59 (±2.09)
SOFA < 9, n (%)	193 (94.6)
BMI, mean (±SD)*	29.45 (±8.29)
18.9-24, n (%)	37 (20)
24.1-29, n (%)	72 (35.3)
30-34.9, n (%)	38 (20.5)
>35, n (%)	38 (20.5)
ICH score, mean (±SD)	2.81 (±1.39)
ICH score, median (IQR)	4 (2-4)
Surgical evacuation, n (%)	19 (9.3)
External ventricular drain placement, n (%)	48 (23.5)
Radiologic characteristics	
ICH location, n (%)	
Basal ganglia	68 (33.3)
Thalamic	35 (17.2)
Lobar	62 (30.4)
Cerebellar	17 (8.3)
Brainstem	13 (6.4)
Multi-territory	9 (4.4)
ICH laterality, n (%)	
Left	94 (46.1)
Right	93 (45.6)
Bilateral	17 (8.3)
ICH volume (in cc), mean (±SD)	48.50 (±46.85)
ICH volume (in cc), median (IQR)	56.67 (11.20-69.88)
ICH volume categorical, n (%)	
<25 cc	87 (43.1)
25-60 cc	51 (25.2)
>60 cc	64 (31.7)
Brain stem component, n (%)	114 (55.9)
Midline shift, n (%)	149 (73)
Hydrocephalus, n (%)	146 (71.6)
Intraventricular hemorrhage, n (%)	140 (68.6)
Mortality characteristics	
Early mortality (N= 109), n (%)	96 (88)
Overall mortality, n (%)	109 (53)
Timing of mortality, in days, mean (±SD)	3.40 (±4.04)
Timing of morality, in days, median (IQR)	2 (1-4)
Cause of death among all deaths (N=109), n (%)	
Brain death	62 (56.9)
Comfort-focused care	44 (40.4)
Unexpected medical	3 (2.8)
Overall outcome	
ICU LOS (in days), mean (±SD)	6.26 (±6.38)
ICU LOS (in days), median (IQR)	2 (2-9)
Hospital LOS (in days), mean (±SD)	11.52 (±15.21)
Hospital LOS (in days), median (IQR)	2 (2-15)

Overall, patient characteristics were comparable between the two cohorts (Table 2) except for the EM cohort, which had a lower proportion of patients with hypertension and end-stage renal disease (ESRD) and lower Glasgow Coma Score (GCS) on admission. This cohort also had a lower proportion of patients who underwent surgical evacuation and EVD placement. In terms of radiographic characteristics, the EM cohort had a higher proportion of lobar hemorrhages, brainstem involvement, midline shift, hydrocephalus, intraventricular hemorrhage, and larger ICH volumes. The ICH score was subsequently higher in the EM cohort.

**Table 2:** Comparison of patient characteristics between early mortality versus survivors among mechanically ventilated patients with spontaneous intracerebral hemorrhage BMI, body mass index; ESRD, end-stage renal disease; GCS, Glasgow Coma Score; ICH, intracerebral hemorrhage; SOFA, sequential organ function assessment

Patient characteristics	Early mortality, N=96	Survivor, N=108	p-Value
Demographics			
Age (in years), mean (±SD)	62 (±15)	58 (±14)	0.058
Female sex, n (%)	53 (55.2)	49 (45.4)	0.161
Race, n (%)			0.289
African American	60 (62.5)	77 (71.3)	
Caucasian	35 (36.5)	29 (26.9)	
Hispanic	1 (1)	0	
Native American	0	1 (0.9)	
Unknown	0	1 (0.9)	
Comorbidity characteristics			
Charlson Comorbidity index, mean (±SD)	2.14 (±1.63)	2.22 (±1.731)	0.714
Charlson Comorbidity index, median (IQR)	2 (1-3)	2 (1-4)	0.828
Comorbidity, n (%)			
Stroke	0	4 (4)	0.057
Intracerebral hemorrhage	0	4 (4)	0.056
Hypertension	65 (67.7)	88 (81.5)	0.023
Coagulopathy	7 (7)	14 (13)	0.183
Diabetes	21 (21.9)	32 (29.6)	0.207
ESRD	8 (8)	20 (18.5)	0.035
Admission clinical characteristics			
GCS on admission, mean (±SD)	4.83 (±3.28)	8.14 (±4.12)	<0.001
GCS on admission, median (IQR)	3 (3-6)	7 (5-12)	<0.001
SOFA, mean (±SD)	5.22 (±1.76)	4.12 (±2.20)	<0.001
SOFA category			
SOFA=9, n (%)	91 (96)	102 (97)	0.603
BMI, mean (±SD)	29.30 (±9.36)	29.57 (±7.39)	0.824
ICH score, mean (±SD)	3.63 (±1.15)	2.10 (±1.17)	<0.001
ICH score, median (IQR)	4 (3-4)	2 (1-3)	<0.001
In-patient surgical procedures			
Surgical evacuation, n (%)	2 (2)	17 (15.7)	0.001
External ventricular drain placement, n (%)	8 (8.3)	40 (37)	<0.001
Radiologic characteristics			
ICH location, n (%)			0.013
Basal ganglia	25 (26)	43 (39.8)	
Thalamic	12 (12.5)	23 (21.3)	
Lobar	32 (33)	30 (27.8)	
Cerebellar	13 (13.5)	4 (3.7)	
Brainstem	9 (9.4)	4 (3.7)	
Multi-territory	5 (5.2)	4 (3.7)	
ICH laterality, n (%)			0.022
Left	38 (39.6)	56 (51.9)	
Right	45 (46.9)	48 (44.4)	
Bilateral	13 (13.5)	4 (3.7)	
ICH volume (in cc), mean (±SD)	69.19 (±55.05)	30.48 (±28.01)	<0.001
ICH volume (in cc), median (IQR)	56.67 (20.06-108.50)	20.60 (8.88-42.45)	<0.001
ICH volume (in cc), minimum (maximum)	0.30 (209.20)	0.09 (107.70)	
ICH volume categorical, n (%)			<0.001
<25 cc	26 (27.7)	61 (56.5)	
25-60 cc	22 (23.4)	29 (26.9)	
>60 cc	46 (48.9)	18 (16.7)	
Brain stem component, n (%)	78 (81.3)	36 (33.3)	<0.001
Midline shift, n (%)	79 (82.3)	70 (64.8)	0.005
Hydrocephalus, n (%)	81 (84.4)	65 (60.2)	<0.001
Intraventricular hemorrhage, n (%)	77 (80.2)	63 (58.3)	<0.001

The multivariate logistic regression analysis, included history of hypertension, history of ESRD, GCS on admission, sequential organ function assessment (SOFA) score, ICH volume, location and laterality, brainstem component, presence of midline shift, hydrocephalus, IVH and EVD placement in the model. The model showed that ICH volume and brainstem involvement increased the odds of EM, while history of hypertension, surgical evacuation, EVD placement decreased the odds of EM (Table 3).

**Table 3:** Multivariate logistic regression analysis of possible predictors of early mortality among mechanically ventilated patients with spontaneous ICH GCS, Glasgow Coma Score; ICH, intracerebral hemorrhage; SOFA, sequential organ function assessment

Variables	p-Value	OR (95% CI)
History of hypertension	0.010	0.246 (0.085-0.712)
History of end-stage renal disease	0.298	0.484 (0.123-1.902)
GCS on admission	0.078	0.872 (0.749-1.016)
SOFA score	0.812	0.968 (0.738-1.296)
ICH volume	<0.001	1.024 (1.012-1.037)
ICH laterality	0.093	1.913 (0.896-4.084)
Brain stem component	0.001	5.660 (2.012-15.917)
Hydrocephalus	0.638	1.401 (0.344-5.698)
Intraventricular hemorrhage	0.977	0.981 (0.270-3.564)
Location	0.574	1.097 (0.0.795-1.514)
Surgical evacuation	0.006	0.044 (0.005-0.412)
External ventricular drain placement	0.001	0.130 (0.040-0.417)

Among all the patients under the EM cohort, the mean time to death was 2.13 (SD ±1.45) days with 62% (59) due to brain death and 37% (35) due to palliative extubation or transition to comfort-focused care (Table 4). Of note, patients who had surgical evacuation included only those with ICHs located supratentorial or in the cerebellum. Brainstem-only hemorrhage patients did not undergo surgery in this cohort [9].

**Table 4:** Mortality characteristics of mechanically ventilated patients with sICH sICH, spontaneous intracerebral hemorrhage

Patient characteristics	Early mortality, N=96	Late mortality, N=13	p-Value
Timing of mortality (in days), mean (±SD)	2.13 (±1.45)	12.85 (±4.51)	<0.001
Timing of mortality (in days), median (IQR)	2 (2)	11 (5)	<0.001
Cause of mortality, n (%)			0.026
Brain death	59 (61.5)	3 (23.1)	
Withdrawal of care	35 (36.5)	9 (69.2)	
others	2 (2.1)	1 (7.7)	

## Discussion

This study aimed to determine the proportion of EM among patients with sICH. The independent predictors of EM in this cohort of patients were also identified. Novel to this study, it was found that EVD placement and surgical evacuation along with a history of hypertension decreased the odds of EM. In this study, the proportion of EM was 47%, which is slightly higher than the previous reports [6,10]. This can be partly attributed to the selection of sickest patients who are MV with sICH in our study. Among all the characteristics, it was found that the ICH volume and brainstem involvement independently increased the odds of EM.

The volume of ICH is the best indicator of EM for any given place in the brain according to a prior study [11]. In a retrospective analysis of 30-day mortality rates based on ICH volume, it was observed that ICH volume of >60 cc was associated with higher mortality rate compared to those <60 cc [12]. In our study, the mean ICH volume for the EM cohort was significantly higher than the survivor cohort. This is consistent with prior reports, which have shown that as ICH volume increased, the patient’s 30-day mortality rate increased [5]. This indicated an impactful value of hemorrhage volume at presentation [5]. Our study reveals that ICH volume is also a significant factor to consider for those patients expiring even before seven days.

Brainstem involvement has been reported to be an independent predictor of mortality in sICH patients [4]. And if primarily involved, it is considered the most fatal location of ICH [13]. It has been reported before that a loss of ≥ 3 brainstem reflexes and the swirl sign on the baseline CT scan can be simple potential prognostic indicators of brain death for patients with ICH [4]. In another study analyzing the predictors of one-month case fatality, it was found that brainstem ICH was an independent predictor [14]. This study has found that the proportion of brainstem involvement was higher in the EM cohort as well. This is consistent with prior studies.

External ventricular placement and surgical evacuation decreased the odds of EM among MV sICH patients in this study. In this institution, these procedures are decided based on shared decision-making among the patient’s family members, the neurocritical care team, the stroke team, and the neurosurgical team, along with the consideration of clinical factors and imaging findings [9]. Considering the mortality of patients at seven days in the minimally invasive surgery with thrombolysis in intracerebral hemorrhage evacuation (MISTIE III) trial, those who underwent surgery versus standard medical group had reduced mortality [15]. Hanley’s et al. study showed reduced mortality in patients who underwent surgery [15]. In another study of patients between 16 and 49 years of age, it was found that surgical evacuation was also associated with a lower odds of mortality [16]. A study on early surgery for supratentorial sICH similarly revealed a lower 12-month mortality rate if surgery was done before or at day 1 [17]. A study analyzing patients with ICH who required an EVD showed that among the patients who required an EVD, for each minute that EVD placement was delayed, the odds of death increased by 0.03% [18], showing that EVD placement is an independent factor that has to be considered to improve mortality in this patient population. Another study indicated that although ICP monitor placement including EVDs has the potential to increase the risk of poor outcome among patients with sICH, this effect does not translate to an increase in mortality [19], which makes EVD placement in sICH still variable among clinicians. These studies are consistent with the findings that surgical intervention and EVD placement have the potential to improve mortality among patients with sICH [15-19]; however, the decision to pursue these remains widely variable. In another retrospective analysis of 30-day mortality in sICH patients, it was found that when compared to patients who received the best medical care, patients who underwent surgical therapy had a trend toward a higher survival rate [20]. These studies are consistent with our findings that surgical evacuation was associated with lower odds of EM when compared to the survivor cohort (2% vs. 15.7%, p<0.001).

History of hypertension was found to be a protective factor in this patient cohort. Although the exact mechanism is unknown, antihypertension medications may provide the pharmacologic protective factor of limiting the expansion of the bleed. Regardless, further studies are needed to explore the proper reasons. An analysis conducted by Al-Khaled et al. identified patients who had a history of hypertension had improved outcomes [21]. Among the 474 patients of that study with a history of hypertension, 374 patients survived sICH and 99 patients died (p = 0.002) [21]. In a study of 370 patients, a history of hypertension was estimated to double the probability of incidence of ICH, (OR = 2.55, 95% CI: 1.72-3.79) [22,23]. Sustained hypertension in the early stages of ICH can create a risk of persistent hemorrhage, increased volume of ICH, and worsening severity of stroke following the initial insult [24]. A 10 mm Hg rise in systolic blood pressure was found in a study by Sakamoto et al. (n=211) to be correlated with poor patient outcomes [25]. However, this analysis showed the EM cohort had a lower proportion of patients with a history of hypertension when compared with the survivors (67.7% vs. 81.5%, p = 0.023).

Mortality characteristics

This study revealed that in this population of young African American patients who are MV, the proportion of those who expired before seven days was 88% of all deaths. The mean time from admission to death was 2.13 (SD ±1.45) days, with the most common cause of death being brain death. Patients from the EM cohort are already sicker with lower GCS on admission in addition to having higher ICH scores, which makes it less likely for these patients to survive without any appropriate neurosurgical intervention. However, in sICH, available neurosurgical interventions such as minimally invasive surgery for supratentorial ICH and IVHs have not been shown to improve patients’ functional outcome [26]. Thus, the decision-making for proceeding with neurosurgical intervention is complex and should be individualized, especially in these patients with poor examination scores on admission.

We have found that in this study, the mortality rate is slightly higher than what has been previously reported in other similar studies [6,10] on EM (31.9-32.7%). Also, the patient population is much younger. Among the EM cohort, the proportion of brain death is higher compared to what has been reported for EM (29.2%) [6]. This cohort is unique in that it is most representative of the young African American population who have severe acute brain injury due to sICH and are on mechanical ventilation. Furthermore, this study shows that there may be potential to consider surgical interventions such as EVD placement in this young group of patients to decrease their odds of death in the first seven days of hospitalization through continued interdisciplinary discussion and shared decision-making with the family. History of hypertension was found to be protective, and these findings need to be explored mechanistically as these patients may have an adaptive cerebral and systemic autoregulatory mechanism that enables them to tolerate this type of severe brain injury.

Limitations

The limitations of this study include its retrospective nature and the modest number of patients in this sample. This study's findings may have been affected by confounding variables such as medical comorbidities, which could have led to the observed differences in the patient outcomes. As medical devices, treatments, and technology have improved dramatically over the past 10 years, these improvements may also have led to the observed patient outcomes. In some of the patients, incomplete data included rescue care and causation of death. The decision for surgery and EVD placement is multifactorial and determined by interdisciplinary discussion as well as shared decision-making processes with the family, which makes it difficult to quantify how these decisions are made. This study included the sickest patients as evidenced by the patients being MV. This patient cohort is largely African American and mostly consists of young patients. They are the population that this study can be generalized to as this population has not been widely studied. Another point to be noted is that the high EM of 47% is among the sickest patients who consist of the entire cohort and should not be generalized to any sICH patient. The major cause of death in this MV patient cohort was brain death.

## Conclusions

This retrospective study of MV patients with sICH treated at a major academic medical center identified an overall mortality rate of 53%, among which 88% were considered EM. Among all the patients under the EM cohort, the mean time to death was 2.13 (SD ±1.45) days, with 62% (59) due to brain death. Among all the characteristics, it was found that the ICH volume and brainstem involvement independently increased the odds of EM, consistent with prior studies. Novel to this study, it was found that EVD placement and surgical evacuation along with a history of hypertension decreased the odds of EM among MV patients with sICH. Further studies are needed to explore potentially modifiable processes that can improve EM and patient outcomes.
